# 

**Published:** 1879-11

**Authors:** 


### BOOKS AND PAMPHLETS RECEIVED.

Transactions of the College of Physicians of Philadelphia. Third series, Vol. IV. 1879. Philadelphia: Lindsay & Blakiston.

Examination of Urine, with Special Reference to the Diseases of the Urinary Apparatus. By K. B. Hoffmann and B. Ultzmann. Second edition. Translated and edited by F. Forchheimer, M.D. Illustrated. Cloth, pp. 195. Cincinnati: Peter G. Thomson.

The Multum in Parvo Reference and Dose Book. By C. H. Leonard, M.D. Cloth, pp. 97. Third edition, revised and enlarged. 1879. Detroit.

Physicians' Pocket Day-Book. By C. H. Leonard.

Transactions of the Medical and Chirurgical Faculty of the State of Maryland. Eighty-first Annual Session. Baltimore, 1879.

The Grounds of a Homœopath's Faith. Three Lectures delivered at the request of Matriculates of the Department of Medicine and Surgery (old school) of the University of Michigan. By S. A. Jones, M.D.

Transactions of the New York Obstetrical Society for the Years 1876–77 and 1878, with a List of the Fellows since its foundation. Reprinted from the *American Journal of Obstetrics*, for private distribution by the Society. Vol. I. New York.

Student's Pocket Medical Lexicon, giving the Correct Pronunciation and Definition of all Words and Terms in General Use in Medicine and the Collateral Sciences, the pronunciation being plainly expressed in the American Phonetic Alphabet; with an Appendix containing a list of Poisons and their Antidotes, Abbreviations used in Prescriptions and a Metric Scale of Doses. By Elias Longley. Cloth, pp. 303. 1879. Philadelphia: Lindsay & Blakiston. Chicago: Jansen, McClurg & Co.

Treatment of Fractures of the Femur. By C. Truesdale, M.D. Reprint from Transactions of Illinois State Medical Society. 1879.

Buildings for Insane Criminals. By Walter Channing, M.D. Read at the Conference of Charities in Chicago, June, 1879.

Address delivered before the New York Auxiliary Sanitary Association. By J. H. Rauch, M.D.

Physiological Antagonism the Therapeutic Law of Cure. By Electus B. Ward, M.D. Read before the Detroit Academy of Medicine, June 10, 1879

Proceedings of the Sixth Annual Meeting of the Oregon State Medical Society, held at Portland, June 12, 13, 1879.

The Hot Springs of Arkansas as a Health Resort. By J. M. Keller, M.D. Reprint from *St. Louis Medical and Surgical Journal*, August, 1879.

Statistics of Placenta Prævia, collected from the Practice of Physicians in the State of Indiana. By Enoch W. King, M.D.

Sanitary Problems of Chicago, Past and Present. By J. H. Rauch, M.D. Reprint from Transactions of the American Public Health Association, Vol. IV.

Thirty-sixth Annual Report of the Managers of the State Lunatic Asylum, Utica, N. Y., for the year 1878.

Cleanliness, Health, Wealth. Report to New Orleans Medical and Surgical Association.

Report of the Special Committee on Medical Education, before the Illinois State Medical Society, at its Twenty-ninth Anniversary, held at Lincoln, May, 1879. Chairman of Committee, E. Ingals, M.D.

Proceedings of the Alumni Association of Rush Medical College, Chicago, 1879.

Address of Dr. C. Goodbrake, president, before the Alumni Association of Rush Medical College, Chicago, 1879. Reprint from the Proceedings of the Alumni Association of Rush Medical College, 1879.

Minutes of the Medical Society of the County of New York, 1806–1878. A. E. M. Purdy, editor. Part V.

The Administration of Chloroform. By Wm. Meacher M.D. Reprint from CHICAGO MEDICAL JOURNAL AND EXAMINER, September, 1879.

Address from the Auxiliary Sanitary Association of New Orleans to the other Cities and Towns in the Mississippi Valley.

Seventh Annual Report of the State Board of Health of Minnesota, January, 1879.

Transactions of the State Medical Society of Kansas, 1879.

Transactions of the Twenty-sixth Annual Meeting of the Medical Society of the State of North Carolina, 1879.

---

THE TREATMENT OF CONVULSIONS IN CHILDREN. (Ferrand. *L'Union Médicale*, Sept. 9, 1879.)

The author advises the bath, bromide of potassium internally, inhalations or chloroform, in case of extreme violence of the convulsions, anointing the axillæ with belladonna ointment. If the indications require action of the bowels, he prescribes calomel in place of the bromide, or alternating with it. In grave forms it is well to apply leeches back of the ears.

### ANNOUNCEMENTS FOR THE MONTH.

### SOCIETY MEETINGS.

Chicago Medical Society—Mondays, Nov. 3 and 17.
West Chicago Medical Society—Mondays, Nov. 10 and 24.

### CLINICS.

MONDAY.

Eye and Ear Infirmary—2 p. m., Ophthalmological, by Prof. Holmes; 3 p. m., Otological, by Prof. Jones.

Mercy Hospital—1:30 p. m., Surgical, by Prof. Andrews.

Rush Medical College—2 p. m., Dermatological and Venereal, by Prof. Hyde; 3 p. m., Medical, by Dr. Bridge.

Woman's Medical College—2 p. m., Dermatological, by Prof. Maynard; 3 p. m., Diseases of the Chest, Prof. Ingals.

TUESDAY.

Cook County Hospital—2 to 4 p. m., Medical and Surgical Clinics.

Mercy Hospital—1:30 p. m., Medical, by Prof. Hollister.

WEDNESDAY.

Chicago Medical College—1:30 p. m., Eye and Ear, by Prof. Jones.

Rush Medical College—3:30 to 4:30 p. m., Diseases of the Chest, by Dr. E. Fletcher Ingals.

THURSDAY.

Chicago Medical College—1:30 p. m., Medical, by Prof. Quine.

Rush Medical College—3 p. m., Diseases of the Nervous System, by Prof. Lyman.

Eye and Ear Infirmary—2 p. m., Ophthalmological, by Dr. Hotz.

Woman's Medical College—3 p. m., Surgical, by Prof. Owens.

FRIDAY.

Cook County Hospital—2 to 4 p. m., Medical and Surgical Clinics.

Mercy Hospital—1:30 p. m., Medical, by Prof. Davis.

SATURDAY.

Rush Medical College—2 p. m., Surgical, by Prof. Gunn.

Chicago Medical College—2 p. m., Surgical, by Prof. Isham; 3 p. m., Neurological, by Prof. Jewell.

Woman's Medical College—11 a. m., Ophthalmological, by Prof. Montgomery; 2 p. m., Gynæcological, by Prof. Fitch.

Daily Clinics, from 2 to 4 p. m., at the Central Free Dispensary, and at the South Side Dispensary.

